# Lessons Learned during a Naturalistic Study of Online Treatment for Pediatric Rehabilitation

**DOI:** 10.3390/ijerph18126659

**Published:** 2021-06-21

**Authors:** Naomi Gefen, Shoshana Steinhart, Maurit Beeri, Patrice L. Weiss

**Affiliations:** 1 ALYN Hospital, Jerusalem 91090, Israel; ssteinhart@alyn.org (S.S.); maurit@alyn.org (M.B.); plweiss@gmail.com (P.L.W.); 2 Department of Occupational Therapy, University of Haifa, Jerusalem 34988, Israel

**Keywords:** COVID-19, pediatric rehabilitation, online therapy, tele-rehabilitation, telehealth, focus group, remote therapy

## Abstract

The COVID-19 pandemic forced many health care providers to modify their service model by adopting telehealth and tele-rehabilitation with minimal time to plan for its execution. ALYN—Pediatric Rehabilitation Hospital in Jerusalem, Israel, responded with alacrity by providing a broad range of rehabilitation services to young people via online therapy during the first 5 months of the pandemic. The objectives of this naturalistic study were: (1) to monitor usage and user experience of online rehabilitation provided to young people receiving out-patient sessions of physical therapy, occupational therapy, speech and language therapy and psychology and (2) to consider the advantages and disadvantages of retaining this model of online treatment in full or in part post-COVID-19. The online rehabilitation treatment program was provided to 147 young people, aged 3 months to 20 years (mean 8.5 y; SD 5.3), and monitored and evaluated via data from the medical records as well as interviews, questionnaires and focus groups. The results use descriptive and inferential statistics to analyze data on the types and frequencies of therapy provided to 147 young people. Over a five month-period, 2392 therapy sessions were provided, 61 therapists from four disciplines were involved and 56.4% of the young people received two or more types of therapies via online rehabilitation. A repeated measures ANOVA showed significant differences over time per therapy. Feedback and recommendations about the process from therapists, parents and young people were collected during two focus groups of the professional staff (*n* = 12), parents and young people (parents *n* = 5, young people *n* = 3). Tele-rehabilitation services were perceived to be beneficial and effective by the great majority of young people, their parents and the healthcare professionals. The results are discussed within the context of conventional therapy as well as in comparison to reports of other online services for similar populations. We conclude that a hybrid approach in which in-person therapy sessions are coordinated with synchronous, online sessions, will provide a best-case fit for young people with chronic disabilities.

## 1. Introduction

Since the early 2000s, emerging developments in online technology have offered increasing opportunities to support remotely delivered healthcare [1,2,3]. Telehealth refers to all health services provided remotely and tele-rehabilitation, more specifically, refers to the use of information and communication technologies (ICT) to provide rehabilitation services to people remotely in their homes or other environments [4,5]. The goal of tele-rehabilitation is to improve client access to care by receiving therapy beyond the physical walls of a traditional healthcare facility, thus expanding the continuity of rehabilitation care [6,7,8,9]. The technology used by rehabilitation professionals is diverse, ranging from simple day-to-day applications such as phone calls, emails, photos or videos, to more sophisticated technologies such as secured video conferencing, sensor-based monitoring, and virtual gaming. These can be used to increase the accessibility and the cost-effectiveness of rehabilitation [10,11].

As demonstrated by numerous systematic reviews and meta-analyses that examined more than 250 studies of tele-rehabilitation published in the past 20 years, the majority have focused on adult populations with diverse diagnoses (e.g., stroke, multiple sclerosis (MS), diabetes, heart failure, chronic obstructive pulmonary disease, cancer) [10,12,13,14]. The studies did not report any negative effects of tele-rehabilitation on the participants or statistically significant differences between conventional care to telehealth. In McFarland’s et al. [14] systematic review and meta-analysis, additional qualitative data were provided. Participants reported peace of mind, reassurance, patient ownership and an overall feeling of better access to health care.

Prior to the COVID-19 pandemic, the use of this approach for pediatric rehabilitation had been reported with far less frequency. Camden et al.’s [3] systematic review summarized the results of 23 studies of children from birth to the age of 12 years including diagnoses such as autism, cerebral palsy (CP), acquired brain injury (ABI) and other developmental disabilities and the effectiveness of tele-rehabilitation. The majority of studies (18/23) were carried out since 2015 and most focused more on behavioral issues than on physical or functional goals. None of the studies reported in this systematic review documented any advantage of in-person sessions over tele-rehabilitation, concluding that, in many cases, tele-rehabilitation appears to be as beneficial as in-person therapy. Indeed, in applications of coaching and behavioral therapy, tele-rehabilitation appeared to be more effective than physical therapy via tele-rehabilitation.

Yet, the use of tele-rehabilitation was not widespread in the great majority of clinical settings until the necessity arose of providing therapy via remote means due to the COVID-19 pandemic. Previously, concerns regarding data security as well as disruption of the patient-clinician relationship led some rehabilitation professionals to resist adoption of these tools [15]. Moreover, many therapists were reluctant to embrace what they perceived to be the burden of coping with online technologies [15,16]. Remote rehabilitation had mainly been used as a means to provide services to those living in the periphery who have less access to services [17]. A systematic review of 44 studies of allied health care professionals providing remote services in Australia between the years 2004–2015 found that the majority concentrated on speech pathology (64%). Thirty-three studies reported on trials and interventions with the remainder focusing on the client’s perception of the service. None of the studies reported a deterioration or negative results, but the authors did report that, in general, there was no noticeable increase in the use of remote therapy and that the therapists remained hopeful that upcoming improvements in technology will improve the use of such services.

Gladwell’s “tipping point” [18] was achieved with the onset of the COVID-19 pandemic crisis where the need to expand the delivery of online pediatric rehabilitation services became of paramount importance. Conventional healthcare services were unable to adequately cope with therapeutic interventions such as medication monitoring and dietary supervision, nor to provide cognitive and motor therapy as well as psychological support [4]. Children with disabilities who did not have acute, life-threatening medical needs were recommended to avoid hospital visits and in-person contact with clinicians; the ability to access therapy remotely became the option of choice, indeed the only available alternative [19].

The approach of the Mayo Clinic team [20] illustrates this response. To prepare children, families and therapists for tele-rehabilitation consultations necessitated by the COVID-19 pandemic social distancing requirements, they developed checklists and guidelines that were specific for medical diagnoses and types of intervention. The checklists included the items that therapists and patients needed to prepare prior to therapy in the home setting such as tools or equipment and any technological requirements for a successful online visit. A wide range of concerns related to how to execute specific assessments and provide treatment in a home setting, the type of equipment needed to record clinical outcomes, and how to build rapport from a distance. They concluded that remote interactions were generally suitable for pediatric rehabilitation services and that the pandemic crisis should be used to “try out” best practices for the future. Tanner [21,22] reported on tele-rehabilitation services provided to children in general and oncology departments; within two weeks of the COVID-19 pandemic outbreak, protocols were developed, and within the first month, more than 1000 tele-rehabilitation sessions were provided.

In summary, the potential of online technologies for pediatric rehabilitation has been demonstrated in a range of clinical interventions with children and their families. They appear to encourage greater physical activity, promote a healthier lifestyle, help manage chronic conditions and, indeed, to be viable alternatives to traditional one-on-one interventions [3,23,24]. Nevertheless, despite initial promising research results, the adoption of these technologies has been limited, and even waned after the first wave of excitement [25]. Additional evidence is required to identify “best practices” for maintaining viable clinician–patient interactions that promote activity and participation, while supporting families in their efforts to manage their children’s health condition and psychological well-being.

In light of the COVID-19 pandemic, ALYN Pediatric Rehabilitation Hospital responded immediately by providing a broad range of rehabilitation services to young people via online therapy. Until that point ALYN provided tele-rehabilitation services only within the framework of pilot studies and research [24]. Widespread reservations among therapists, reimbursement difficulties and lack of Health Insurance Portability and Accountability Act (HIPAA) [26]-accredited infrastructure prevented the inclusion of tele-rehabilitation into the regular program offered by the hospital. However, once COVID-related health regulations forced abrupt cessation of on-site ambulatory services and brought treatment protocols to a halt, every single one of these barriers was quickly overcome. Similar to other countries, the Israel Ministry of Health (IMOH) issued the needed provisions, recognizing tele-rehabilitation therapy as equivalent to in-person sessions reimbursement-wise. This paved the way to a combination of on-site and off-site patients and therapists. For example, in-patients could be treated remotely by therapists who were either quarantined themselves or in lock-down, and ambulatory multidisciplinary clinics could be held as long as enough data could be obtained without physical examination.

Within one week of the IMOH’s regulations mandating an enforcement of social distancing, ALYN responded by implementing medical and allied health online support including speech and language, physical and occupational therapy, psychology treatments and more. In keeping with the well-known adage “Necessity is the mother of invention”, our goal was to implement the use of a HIPAA accredited online platform (Zoom) that is widely available, user-friendly and that supports the implementation of realistic therapy protocols via real-time interactions between one or more patients and therapists.

For young people in the critical period of rehabilitation, such as those recovering from brain injury or following orthopedic and neurosurgical interventions, Zoom-based online therapy was adopted without delay by ALYN, provided that the young people’s parents or guardians consented in writing to this method. At the same time, the hospital administrators, clinicians and research personnel made a key decision to fully document the process in order to determine its feasibility, effectiveness and acceptance by the various stakeholders. Thus, although the adoption of online therapy was an ad hoc response to a crisis, documentation of the experience was expected to support future decision- making regarding the advantages and limitations of its continued use after COVID-19. We expected online therapy monitoring would provide information as to which patients and treatment goals are most amenable to remote interaction, and that we could use the data to develop patient selection criteria. An additional goal included identification of appropriate treatment goals that take into account the flexibility needed in treatment approaches since, in some cases, online tasks differ greatly from those suitable for conventional in-person therapy. Thus, the objectives of this naturalistic study were: (1) to monitor usage of and attitudes toward online rehabilitation provided to young people receiving out-patient sessions of physical, occupational, speech and language therapy and psychology and (2) to determine whether this model of online treatment should be retained in full or in part post-COVID-19.

## 2. Materials and Methods

### 2.1. Research Design

This was a prospective study that aimed to document an ongoing online clinical intervention. A mixed model (quantitative and qualitative methods) based on subjective and objective data from a large convenience sample of participants was used. Outcomes from each participant were followed over the first five months of the hospital’s adoption of online therapy as well as grouped and compared based on the treatment type and objective, severity of condition, and age. This approach is based on a “naturalistic” approach to data collection necessitated under the conditions of realistic clinical practice [27]. Behaviors or other phenomena of interest were observed in their natural settings without manipulation of any kind and recorded by the researcher.

### 2.2. Participants

This study consisted of three populations: the young people in treatment at ALYN (*n* = 147) age 3 months to 20 years old (mean 8.5 y; SD 5.3 y), their parents (and other caregivers) who facilitated the process, and the therapists who provided the treatment. Patients were selected by their accessibility to the relevant technology and their ability and willingness to cooperate and, in cases where the patient was very young, or with limited intellectual ability or severe motor disabilities, the parents’ ability to receive and implement the instruction. The young people included post-orthopedic surgery patients at the sub-acute stage, some post-surgical with underlying neurological disorder, as well as young people undergoing longer-term treatment plans as day-patients such as young people with cerebral palsy, myelomeningocele, arthrogryposis, conversive disorders, complex pain syndromes, complex feeding disorders, acquired brain and spinal injury. Ethical permission for the research was obtained from Institutional Review Board of the ALYN Hospital. Parents signed a consent form and children gave their verbal assent.

### 2.3. Instruments

#### 2.3.1. Data

Personal (age, gender, type of school, religion, number of siblings) and medical data (diagnosis, time after onset, types of therapies received) about each participant were collected and stored in a shared file on the hospital server.

#### 2.3.2. Protocols

Each of the four participating departments (physical therapy, occupational therapy, speech therapy, and psychology) prepared a structured usability protocol which they (the therapist) used to document what took place throughout each session.

#### 2.3.3. Two Focus Groups Were Performed

The first was with representatives from four of the participating departments and additional stakeholders from the hospital (physicians and researchers). The second focus group included young people and parents who participated in remote therapy sessions, a representative of each department and researchers. The purpose of the focus groups was to lead to the formulation of a “best practices” model for subsequent implementation.

#### 2.3.4. Thematic Analysis

A thematic analysis is a way to analyze qualitative data by looking at people’s experiences, opinions or views systematically. The data is collected for example through interviews, surveys or focus groups [28]. This was performed to identify recurring themes from therapists, parents and young people as presented during the focus groups.

#### 2.3.5. Strengths, Weaknesses, Opportunities and Threats Analysis (SWOT)

A SWOT analysis is a method designed to support strategic planning of projects [29]. It is typically initiated by specifying the objective of a particular project and then identifying the internal and external factors that support or detract from achieving it.In our case, a SWOT was used to map the strengths, weaknesses, opportunities and threats identified by therapists, parents and young people following their participation in online therapy based on the topics that we identified during our thematic analysis of the transcribed focus group sessions.

### 2.4. Procedures

#### 2.4.1. Treatment Sessions

All parents of young people who were candidates for online therapy were asked to sign a form confirming their agreement that their child would receive treatment via Zoom or telephone interactions during the acute period of the pandemic related restrictions. All were assured that their refusal to give consent to the treatment would in no way affect their child’s ability to continue in conventional therapy once a return to the hospital was allowed. The duration of each session was 30–45 min with a frequency of 1–2 times weekly. Therapeutic goals included (i) continuing previously set objectives to advance functional goals for each patient, (ii) monitoring the progress of home exercise programs, (iii) modifying the exercises according to patient progress and adapting to home environment and (iv) maintaining goals achieved thus far in therapy until able to return to an on-site therapy program. Treatment continued until goals were met or until a return to the hospital was allowed. At the end of treatment sessions therapists documented their experience.

#### 2.4.2. Therapist Focus Group

The objective of this focus group was to provide a discussion forum of representative therapists from ALYN’s departments who had been participating in online tele-rehabilitation. The focus group convened 8 weeks after starting tele-rehabilitation. The main goals were to gain insights into the relative successes and limitations of online therapy and to determine whether ALYN should continue with it as currently conducted or with significant changes beyond the COVID-19 era. A group of therapists, representatives of the four participating departments and other hospital stakeholders (physicians and researchers) participated in a two-hour video conference focus group, via the Zoom application.

Following a short explanation of goals and agenda, questions were presented to the forum for discussion. The questions included: (1) How do we define success in an online treatment? (2) What contributed or detracted to/from this success? (3) What are some “tips and tricks” to making effective sessions? (4) What is the burden on the therapist, compared to conventional therapy? (5) What would have made this experience more feasible and positive for you? (6) To what extent has this experience been worthwhile for the young people/parents/therapists? (7) Should ALYN continue to provide online therapy? (8) To what extent do you think that a hybrid approach (online combined with in-person) would be best? If so, how to accomplish this (e.g., ratio)? The meeting was recorded and later transcribed for analysis and extraction of main themes.

#### 2.4.3. Parents and Young People Focus Group

The objective of this focus group was to provide a discussion forum of parents and young people who had been participating in online tele-rehabilitation. The main goals were to gain insight into their experiences and to see if similar themes appeared in both groups. Following a short explanation of goals and agenda, questions were presented to the forum for discussion. The questions referred to their experience, enjoyment, satisfaction, usability (the technology), preferences, if they felt that they were progressing in their rehabilitation program, and if they felt an intrusion to their personal space. The forum was recorded and later summarized for analysis and extraction of main themes.

### 2.5. Data Analysis

Descriptive statistics were used to characterize the variables related to the patients, families and therapists as indicated above (counts and percentages for categorical data; means and standard deviations for continuous data). A repeated measures ANOVA was used to test the change in the amount of therapy sessions across time followed by simple effects analysis. Greenhouse-Geiser correction was applied when the sphericity assumption was violated and Bonferroni correction was applied for effect of multiple testing. *p*-value ≤ 0.05 was considered statistically significant. All quantitative analyses were performed using the SPSS v.25 software (IBM Corp. Released 2017. IBM SPSS Statistics for Windows, Version 25.0. Armonk, NY: IBM Corpcompany). Thematic and SWOT analyses of the focus groups were performed to gain a deeper understanding of the advantages and limitations of online therapy.

## 3. Results

### 3.1. Demographic Results

**Figure 1:**
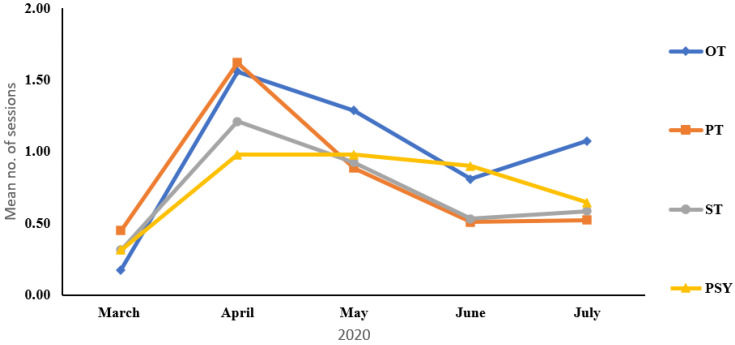
Average therapy sessions per type of therapy over 5-month time period.

Demographic results of patients are presented in Table 1. Sixty-four (43.5%) patients received only one type of online therapy compared to 83 (56.5%) that received two or more types of online therapies. Nine in-patients received online therapy compared to 138 children and young people who received therapy sessions as outpatients. Table 2a,b summarizes the number of online therapy sessions, patients that received sessions and the number of therapists involved in online therapy. In Mid-March 2020 ALYN Hospital started with 184 sessions and by the end of April 2020 the number spiked up to 789 sessions total during the month. By July 2020, the number was down to 416, once full lock-down ended and patients could return to in-person therapy. In order to test the changes in the amount of online therapy session across time, a repeated measures ANOVA was performed that tested these changes over all and within each therapy session type. The analysis revealed a main effect for time (F(3,461) = 48.40, *p* < 0.001), indicating that the mean number of therapy sessions increased sharply between March 2020 and April 2020 and then declined slowly by July 2020, but still remained significantly higher, compared to March 2020 (Figure 1). A significant time X treatment effect was also found (F(8,1131) = 5.58, *p* < 0.001), indicating different slope patterns with each therapy session type: all therapy session types showed a steep rise between March 2020 and April 2020; however, while PT and ST sessions declined between April 2020 and June 2020 and were asymptotic between June 2020 and July 2020, OT sessions increased again between these months, and PSY therapy sessions started to decline only between June 2020 and July 2020 (Figure 1; Table 3).

### 3.2. Thematic and SWOT (Strengths, Weaknesses, Opportunities, Threats) Analysis

In the two focus groups five main topics were identified independently by two of the authors (NG, PLW) with a discussion of discrepancies until consensus was reached and validated by the other two authors (SS, MB) [30]: (1) Technical issues (e.g., regarding ease of use, learning curve of therapists and patients); (2) Continuity of rehabilitation process (e.g., challenges in developing rapport compared to in-person sessions); (3) Physical setting of focusing on available resources and where therapist or child were (home or hospital) (e.g., safety of setting, available therapy modalities); (4) Collaboration with other therapists/parents/young people (e.g., lack of “water fountain” time) and (5) Boundaries (between patients and therapists, between work hours and non-work hours). Multiple participants in both groups raised these themes.

**Figure 2:**
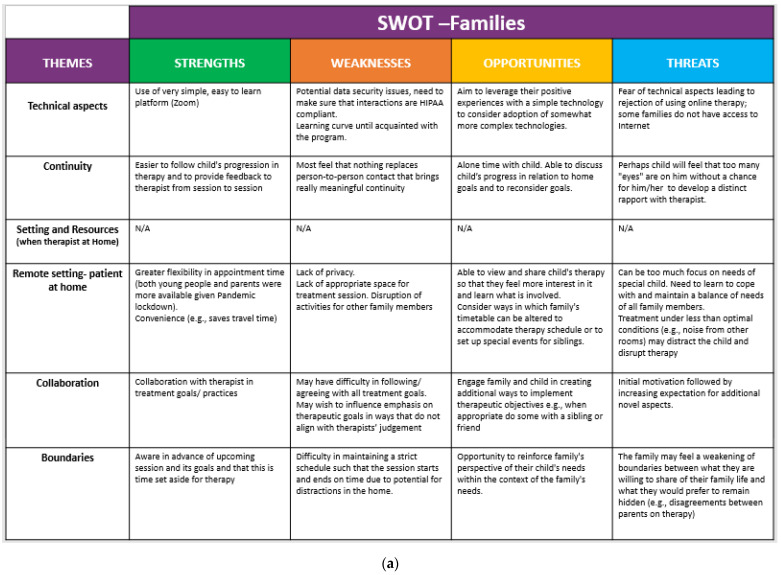

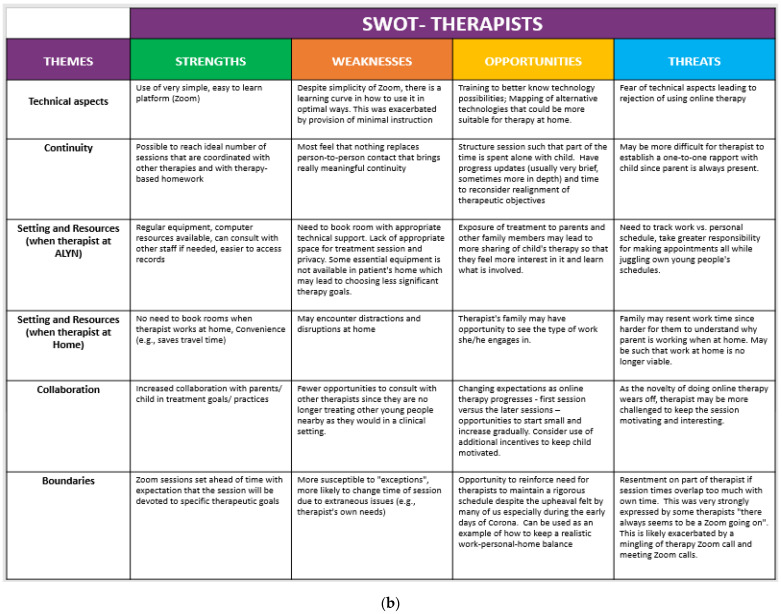
(**a**,**b**) SWOT Analysis-Families and therapists. (**a**) SWOT Analysis-Families. (**b**) SWOT Analysis-Therapists.

The five topics identified in the thematic analysis were further analyzed according to the strengths, weaknesses, threats and opportunities according to the therapists and parents/young people’s point of view as reflected in the different focus groups. It is interesting to note that there were many areas of agreement between both groups (e.g., simplicity of use of Zoom program, use of a hybrid approach wherein the child can receive both in-person and remote therapy) and few areas of disagreement (scheduling that may be better for child/parent potentially collides with the therapist’s own family’s needs). For example, technical strengths by both therapists and families included “use of very simple, easy to learn platform (Zoom)”. On the other hand, with respect to weaknesses, the families reported “an even steeper learning curve due to less practice than therapists had who used it for meetings.” A serious technical threat was related to a “lack of Internet access or willingness to use video conferencing tools” by the more religious families. Nevertheless, we identified the “aim to leverage their positive experiences with a simple technology to consider adoption of somewhat more complex technologies. The results for all five themes are summarized in Figure 2.

## 4. Discussion

The demographic and statistical results highlighted differences over time that varied significantly across therapeutic disciplines: following the sudden significant surge between March 2020 to April 2020 of all therapies, the disciplines responded differently. PT and ST similarly showed an initial increase and then decline in the use of online therapy. The characteristics of therapy, with the desire for more intensive handling (the PTs were eager to position and support the child and stimulate balance reactions; the STs felt the need to provide hands-on oral-motor stimulation) led to the return of patients as soon as possible. Online therapy sessions in psychology remained high until June 2020, due to the primary reliance on verbal interactions with less need of physical contact. Indeed, the psychologists reported that online therapy was, in many cases, more beneficial by facilitating flexibility which led to increased parental involvement in the process. Occupational therapy was the only discipline that decreased between April to June 2020 and then increased during July 2020. This is attributed to the reluctance of parents to send children back to the hospital school; the majority of children in the school are respiratory dependent and need mainly OT and PSY (not PT).

The ensuing discussion will relate to two main topics that emerged from the literature review and study results from global, national and local perspectives: (1) the use of telehealth and tele-rehabilitation before and during the COVID-19 pandemic and 2) “lessons learned” to work towards implementation of “best practices” of tele-rehabilitation. This topic will be examined with reference to a framework for implementing telehealth in pediatrics, known by the acronym VIRTUAL (for Viewing, Information, Relationships, Technology, Unique, Accessible, Legal), proposed by Camden and Silva [11] following a May 2020 e-Summit.

Prior to the COVID-19 pandemic, telehealth was not widely used in pediatric rehabilitation settings despite numerous studies supporting its use as a feasible alternative to conventional in-person healthcare [3,31,32]. A World Health Organization (WHO) report “Global diffusion of eHealth” from 2016 [33] documented the use of telehealth services in different countries based on a survey sent to their member states. One hundred and twenty-two countries (out of 194) responded, with only 22% of the countries reporting the use of telehealth services. In a similar report, Camden and Silva [11] surveyed 76 countries in 2019 and found that only 4% used telehealth prior to COVID-19. It appears that technical, logistical and training challenges deterred therapists from using tele-rehabilitation. However, in May 2020, just three months since the global awareness of COVID-19, the same polled countries reported an increase of 70% [11].

In the United States, extensive use of telehealth was hindered by restrictions related to state-to-state reciprocity in professional licensing and medical insurance coverage [34]. In 2010 only 12 states allowed insurance coverage for these services compared to 2017 where 33 states and the District of Columbia passed laws to enable adoption of telehealth services [35]. The American Academy of Pediatrics recognized that each state and specialty addressed telehealth differently and in 2015 initiated Supporting Pediatric Research on Outcomes and Utilization of Telehealth (SPROUT), a multi-centered research network devoted to establishing evidence for pediatric telehealth. Their first report mapped out telehealth services based on survey responses from 52 programs in 30 states [35]. Considerably more telehealth programs were available in neurology, psychiatry and cardiology (23–25/52) compared to rehabilitation (3/52). The most substantial barrier to growing pediatric telehealth and tele-rehabilitation among the 52 surveyed programs was reimbursement (30/52).

Similar to global trends, Israel also changed its use of telehealth services. A study carried out by a pediatric rehabilitation hospital in Israel [23] demonstrated how the number of tele-rehabilitation sessions increased to 110 over a five-week period in late March to the end of April 2020. They reported that although both children (mean age = 11 years) and therapists were able to maintain a therapeutic “connection” during this period, other rehabilitation goals were perceived to be less achievable, specifically in physical and occupational therapy. Several barriers affected the process including a decline in the children’s focus and attention, problems with technology, and a distracting home environment.

Prior to the COVID-19 pandemic, the experience at rehabilitation facilities around the world and in Israel had, in many cases, supported telehealth services via pilot studies [24]. However, previous concerns regarding the feasibility and acceptance of tele-rehabilitation changed dramatically in many locations, including at ALYN Hospital, as rehabilitation professionals around the world responded rapidly in March 2020 and overcame many barriers. The most prominent barriers were therapist’s hesitation to adopt technology, funding through the health management organizations (HMO) and a concern that many key therapeutic goals could not be obtained.

In 2020, an “info-demiological” evaluation [36], calculated the Relative Search Volume (RSV) and information and the Communications Technology Infrastructure Availability index (ICT) from the World Economic Forum report for the 50 countries most effected by COVID-19. The RSV was calculated by aggregating the information collected from each country, either through Google Trends or the local search engine in Russia and China. Search terms were defined for each of the 50 countries in English, Spanish and the local language. RSV levels were also calculated according to the countries COVID-19 timeline (China January 2020 and all other countries 11 March 2020, in accordance with the WHO announcement of COVID-19 as a pandemic). Although such searches do not necessarily translate to the utilization of telehealth services, they do reflect its perceived significance in each country. Countries with the highest infection and death rates had the highest RSV levels. RSV levels decreased in June and July 2020 (periods with fewer lockdowns) but remained somewhat higher than pre-COVID-19.

The key issue of relevance is whether the tele-rehabilitation experience imposed due to COVID-19 can be harnessed to support future implementations of this approach. Camden & Silva’s [11] VIRTUAL framework, proposed following the May 2020 eHealth Summit for Pediatric Therapists, provides a convenient tool for interpreting the data presented in this paper. In the context of the ALYN experience, *viewing* relates to the insights that the therapists gained by becoming more aware of the patient’s home setting; this can help in modifying the environment, for example, for better training opportunities and compliance. At the beginning of the therapy process, the child or parent provided a virtual tour of the home environment so that an appropriate treatment setting could be selected. Most focus group therapists and parents commented that the opportunity to view the child’s home environment was beneficial in designing between-treatment session exercises that were best suited to overcome setting-specific constraints (e.g., safety concerns in small dwellings). Indeed, this approach is now used even in the case of in-person therapy in order to ensure continuity of therapeutic goals, treatment protocols and home exercise. These findings are in line with the results of Camden et al.’s systematic review [3] of tele-rehabilitation for young people with disabilities and effective intervention characteristics.

*Information* relates to how we provided the information (regarding the therapeutic process) to the patients and families with respect to their preferences and technological abilities. The focus groups related to the benefit of having both parents present when sharing therapeutic information as was often the case during the pandemic. Information was also received from the parents and young people as to how to improve the tele-rehabilitation process. For example, in the second focus group, teenagers participating in online therapy said they preferred to have therapy early in the morning or later in the day so that they did not miss scheduled school sessions via Zoom. Another aspect that emerged in the therapist’s focus group was the challenge in sharing information about the patient between therapists to benefit the overall therapeutic process. Many therapists were providing therapy while they themselves were at home and did not meet the other therapists during breaks. Pre-COVID, a lot of key information was shared between therapists during those breaks, information that was not necessarily documented in the electronic medical record. Similar to other global and local tele-rehabilitation settings [22,23] particular attention is now focused on how information is documented and shared between therapists and with families to better meet their needs for both remote and in-person therapy.

*Relationships* were very important to facilitate the tele-rehabilitation process. Therapists stated that having initial in-person time with the young people provided a better base for the development of the relationship during remote sessions, compared to their connection with patients that they never physically met. In both focus groups, participants related to the difference between a therapy session with a therapist/child they had worked prior to the pandemic. Parents of young people in ALYN’s daycare shared the challenge they faced when participating in therapy- how their role in the triad relationship changed from an observational position to a more “hands on” position. Their role as a parent/ therapist changed the dynamics of the relationship. Parents that were primarily involved in the care for the child with special needs also commented on the impact of this on their relationships with other family members. This aspect remains a major goal for implementing future tele-rehabilitation programs. Other tele-rehabilitation settings also reported the patient-therapist rapport challenge. In some cases [23], therapists noted a positive effect between prior acquaintanceship and online treatment effectiveness. In other cases [17] satisfaction with the tele-rehabilitation services was reported even when the child-clinician relationship developed only during tele-rehabilitation sessions.

*Technology* options were explored by the ALYN information technology team to identify the best option that complied with IMOH’s security regulations. As a hospital, strict regulations were needed, although some of the children were in the hospital’s daycare program, where less strict regulations were required. It was a challenge to match the technology to the patients’ *unique* backgrounds. Due to the moderate number of patients from families without access to the Internet or smartphones, traditional technology such as phone calls were used in some cases. In other cases, at the onset of the pandemic, a simple text, phone and video application was used with the younger children, although it was not approved by the IMOH. As soon as feasible, these patients transferred to the approved technology. The therapists used Zoom functions to facilitate the therapeutic process. For example, “remote control” was granted to the children during therapy. The goal was two-fold: first, to enable interaction similar to that experienced when interacting in-person; second, to give the child some mastery over their own actions during such a restrictive period. Some of the second focus group participants (parents and young people) shared their preference for the familiar technology, such as WhatsApp, LLC, and others indicated that Zoom was too difficult to operate. Going forward, ALYN is considering additional, user-friendly online platforms to better serve the patients and their families during tele-rehabilitation services. Wiederhold [37] remarked on the ramifications that COVID-19 has had on at-home technology usage, for both work and recreational purposes. Whereas approximately 10 million people regularly attended meetings held on Zoom in 2019, this number had greatly expanded to 300 million by April 2020. Although far from ideal, communication technologies such as Zoom supported the continuation of many work-related activities, including rehabilitation, during the quarantine.

One of the key limitations in wider use of online, remote technologies is the lack of inexpensive tools that can provide data comparable to the types of objective, quantitative outcomes needed by some of the healthcare professions. This was particularly noted by the physical therapists who lacked the ability to assess joint range of motion and muscle strength as well as a range of functional abilities such as gait, posture and balance via online treatment. Indeed, the insufficient availability of reliable and valid high-tech devices (e.g., joint position sensors, force transducers) reduce the type of assessments that can be performed via tele-rehabilitation [3,38]. Although still limited, technologies in this field are rapidly developing as demonstrated by tools such as TytoCare, a remote device consisting of a camera, microphone, screen and wireless communication unit. It is equipped with an infrared basal thermometer, digital stethoscope, digital otolaryngoscope and tongue depressors [39]. Although not designed to provide data to support tele-rehabilitation, TytoCare demonstrates the ability of a small, inexpensive medical device to support healthcare providers in the diagnosis and treatment of common ailments (e.g., ear infections, sore throats, fever, allergies, pink eye, nausea, respiratory infections, and common skin conditions) [40]. Meditouch’s ArmTutor and 3D Tutor systems provide feedback of elbow range of motion to treat upper limb function post-elbow fractures via a tele-rehabilitation protocol similar to conventional rehabilitation [41]. The hardware includes wireless electro-optical sensors which detect and display joint movement. The system measures passive and active ROM and movement accuracy in relation to presented virtual targets. It is anticipated that both observational and quantitative assessment of joint range of motion and muscle strength will improve within the next two to five years [42,43].

*Access* to in-person services was limited due to a series of COVID-19 lockdowns. Although patients could return to in-person therapy by July 2020, some families preferred to continue online sessions because of the ease of access to therapy without the need to physically come to the hospital. ALYN continued to provide access to these services when it was apparent that some of the families needed to stay home (e.g., to care for their other children) or because they were afraid to take their respiratory dependent child out of the house. ALYN recognized that some families had access issues and facilitated a donation of computers and game consoles through Microsoft, Inc. [44] to ensure that the children’s therapeutic process would not be interrupted. Parents in the focus group shared that they appreciated that access to therapy was not discontinued. ALYN is committed to providing access to therapy for all patients as an in-person service or online therapy. In countries such as Australia, with many rural areas, access to tele-rehabilitation services is a challenge regardless of COVID-19 [17] and they had already accumulated considerable practice in using these tools. Nevertheless, even for such experienced users, lessons were learned from the world’s response during COVID-19.

Finally, *legal* aspects of tele-rehabilitation were addressed at the commencement of the process and covered privacy and medico-legal aspects. In a systematic review [45] of 30 articles on barriers to the adoption of telehealth services, 33 barriers were identified. Legal liability, security and privacy concerns comprised 11 percent of the barriers identified. In March 2020, the only national policy to substantiate the legal stance of tele-rehabilitation at ALYN hospital was the IMOH published guidelines from 2019 (IMOH 6/2019 directive). The guidelines established that the Israel’s four main HMOs would share responsibility for defining best practices, training and insurance coverage. It also stated that citizens had the right to refuse telehealth services, and that the HMOs could not decrease conventional, in-person services. During the pandemic, in-person services were seriously limited by the HMOs. At the onset of the pandemic, all families under treatment at ALYN were asked if they wanted tele-rehabilitation services. Only those that signed a consent form received services. For others, treatment was stopped abruptly. Some families stated that they wanted to wait and see how long the lock-down would last. After a month, ALYN reached out again to the families that originally declined tele-rehabilitation service. A large number of families decided to permit their children to commence online therapy.

In June 2020, in response to the pandemic and as part of the hospital’s experience, hospital representatives participated in the writing of a position paper on telehealth services in occupational therapy [46]. The paper was accepted by the IMOH. It outlined how remote occupational therapy services should be rendered. For example, choosing the therapy setting at home, parent’s/care giver’s role, security regulations and ethical dilemmas posed to the team during tele-rehabilitation. The IMOH further updated their guidelines in December 2020 (direct communication from IMOH to HMO’s and hospital directors) with specific instructions about reimbursement and consent to online therapy. Further lessons are being documented through IMOH’s special telehealth interest groups including a group of allied health professional working with children with special needs. ALYN has recently updated its policies to include a tele-rehabilitation protocol based on the IMOH occupational therapy position paper [43]. Some countries (e.g., the USA, Australia) had policies on telehealth services in place pre-COVID-19. For example, the American Telemedicine Association has practice guidelines for remote care on a range of different topics including rehabilitation, mental health and dermatology.

### Limitations

It is important to recognize the limitations in this study. As a naturalistic study that started immediately with the onset of the online therapy sessions due to COVID-19, the participants could not be divided into cohorts to compare online and in-person therapies. Data were collected as part of the therapy sessions. In most cases, standard clinical assessments during online sessions could not be performed due to the lack of reliability and validity of these tools for online use. Evaluation of any improvement of function was therefore achieved only through observation of the participant. Future studies of online therapy need to include baseline evaluations, online interventions and follow-up evaluations.

## 5. Conclusions

This study was developed as a naturalistic study that followed the processes that were being developed ad-hoc due to the urgent need for remote therapy created by the pandemic. The technology platforms used were standard, off-the-shelf programs that had their limitations (e.g., due to security issues, sessions not recorded for later analysis). The main limitation was rapid transition to tele-rehabilitation which resulted in insufficient training of the therapists in the use of remote platforms and modification of in-person treatment protocols for remote usage. The therapists were expected to learn the programs independently (with only modest IT support) and to try to achieve the potential of remote therapy. Despite its limitations, the patients received professional services and their therapeutic goals were mostly attained. ALYN Hospital has continued to use tele-rehabilitation since March 2020 in all disciplines mentioned in the study albeit at a reduced rate. Plans to adopt a hybrid approach with therapy sessions in-person and by remote means when appropriate are currently underway. These services will be monitored to compare the feasibility and effectiveness of tele-rehabilitation versus conventional in-person therapy in specific clinical disciplines as well as between disciplines. Input from parents and tele-rehabilitation participants will be a key element towards developing optimal hybrid programs tailored to each individual’s needs as we focus on achieving a better understanding of the tele-rehabilitation experience by all stakeholders.

## Figures and Tables

**Table 1:** Demographic information of participants.

Gender	Girls	71 (48%)				
	Boys	76 (52%)				
Age (years)	Mean: 8.5	SD: 5.3	Range: 3 months–20 years			
Diagnosis	CP: 32 (21%)	Neuromuscular: 20 (13%)	SCI: 9 (6%)	ABI: 12 (8%)	Oncology: 7 (4%)	Other: 67 (45%)
Number of patients that received therapy per discipline	OT: 71 (48%)	PT: 101 (68%)	ST: 61 (41%)	PSY: 42 (29%)		
Number of patients that received therapy per department	In-patient: 9 (6%) Out Patient: 138 (94%)					
Number of young people with just one type of therapy	OT: 4 (2%)	PT: 27 (18%)	ST: 10 (7%)	PSY: 23 (16%)		
Number of types of treatment (OT, PT, ST, Psych)	2 types: 24 (16%)	3 types: 48 (33%)	4 or 5 types: 11 (7%)			

SD = Standard deviation, CP = Cerebral Palsy, SCI = Spinal Cord Injury, ABI = Acquired Brain Injury, OT = Occupational Therapy, PT = Physical Therapy, ST = Speech Therapy, PSY = Psychology.

**Table 2:** (**a**) Number of patients (*n*) that received therapy per discipline and number of online therapy sessions with %. (**b**) Number of therapists that performed online therapy.

(**a**)					
**Number of Online**	**OT**	**PT**	**ST**	**PSY**	**Total**
**Therapy Sessions**					
March	*N* = 23	*N* = 50	*N* = 35	*N* = 20	*N* = 136
	26 (14.2%)	66 (35.8%)	46 (25%)	46 (25%)	184 (100%)
April	*N* = 70	*N* = 95	*N* = 60	*N* = 33	*N* = 285
	229 (29.1%)	238 (30.2%)	178 (22.5%)	144 (18.2 %)	789 (100%)
May	*N* = 60	*N* = 62	*N* = 56	*N* = 36	*N* = 237
	189 (31.5%)	130 (21.7%)	136 (22.7%)	144 (24.1%)	599 (100%)
June	*N* = 47	*N* = 45	*N* = 32	*N* = 34	*N* = 177
	119 (29.5%)	75 (18.5%)	78 (19.4%)	132 (32.6%)	404 (100%)
July	*N* = 49	*N* = 35	*N* = 35	*N* = 24	*N* = 177
	158 (38.1%)	77 (18.5%)	86 (20.6%)	95 (22.8%)	416 (100%)
Total	721	586	524	561	2392
(**b**)					
**Number of Therapists**	**OT**	**PT**	**SP**	**PSY**	**Total**
March	10	17	11	8	46
April	14	25	11	11	61
May	13	17	11	9	50
June	11	12	10	11	44
July	16	13	12	7	48

OT = Occupational Therapy, PT = Physical Therapy, SLP = Speech and Language Pathology, PSY = Psychology.

**Table 3:** Mean, Standard Error (SE) and 95% Confidence Intervals (CI) for each treatment type across time.

Session Type	Month	Mean	SE	95% CI	
**OT**	March	0.18	0.04	0.11	0.25
	April	1.56	0.18	1.20	1.91
	May	1.29	0.17	0.94	1.63
	June	0.81	0.13	0.55	1.07
	July	1.07	0.18	0.71	1.44
**PT**	March	0.45	0.06	0.34	0.56
	April	1.62	0.14	1.34	1.89
	May	0.88	0.10	0.68	1.09
	June	0.51	0.07	0.37	0.65
	July	0.52	0.09	0.35	0.70
**ST**	March	0.31	0.05	0.21	0.41
	April	1.21	0.15	0.91	1.51
	May	0.93	0.13	0.68	1.17
	June	0.53	0.10	0.33	0.73
	July	0.59	0.11	0.38	0.79
**PSY**	March	0.31	0.07	0.17	0.46
	April	0.98	0.20	0.59	1.37
	May	0.98	0.19	0.60	1.36
	June	0.90	0.19	0.52	1.28
	July	0.65	0.15	0.36	0.94

## Data Availability

The data presented in this study are available on request from the corresponding author.

## References

[1] Heinzelmann P.J., Williams C.M., Lugn N.E., Kvedar J.C. (2005). Clinical outcomes associated with telemedicine/telehealth. Telemed. J. E-Health.

[2] Hall M., Lusk K. (2018). Telemedicine. Hum. Ecol..

[3] Camden C., Pratte G., Fallon F., Couture M., Berbari J., Tousignant M. (2019). Diversity of practices in tele-rehabilitation for children with disabilities and effective intervention characteristics: Results from a systematic review. Disabil. Rehabil..

[4] World Health Organization (2020). Implementing Telemedicine Services During COVID-19: Guiding Principles and Considerations for a Stepwise Approach.

[5] Rogante M., Grigioni M., Cordella D., Giacomozzi C. (2010). Ten years of tele-rehabilitation: A literature overview of technologies and clinical applications. NeuroRehabilitation.

[6] Brennan D., Tindall L., Theodoros D., Brown J., Campbell M., Christiana D., Smith D., Cason J., Lee A. (2010). A blueprint for tele-rehabilitation guidelines. Int. J. Tele Rehabil..

[7] Edwards-Gaither L. (2018). Cultural Considerations for Telepractice: An Introduction for Speech-Language Pathologists. Perspect. ASHA Spec. Interest Groups.

[8] Cason J., Hartmann K., Crutchley S. (2016). Use of Telehealth in Early Intervention and School System Practice.

[9] Myers K., Nelson E.L., Rabinowitz T., Hilty D., Baker D., Barnwell S.S., Boyce G., Bufka L.F., Chui L., Comer J.S. (2017). American telemedicine association practice guidelines for telemental health with children and adolescents. Telemed. E-Health.

[10] Johansson T., Wild C. (2011). Tele-rehabilitation in stroke care--a systematic review. J. Telemed. Telecare.

[11] Camden C., Silva M. (2020). Pediatric telehealth: Opportunities created by the COVID-19 and suggestions to sustain its use to support families of children with disabilities. Phys. Occup. Ther. Pediatrics.

[12] Hanlon P., Daines L., Campbell C., McKinstry B., Weller D., Pinnock H. (2017). Telehealth interventions to support self-management of long-term conditions: A systematic metareview of diabetes, heart failure, asthma, chronic obstructive pulmonary disease, and cancer. J. Med. Internet Res..

[13] Amatya B., Galea M.P., Kesselring J., Khan F. (2015). Effectiveness of tele-rehabilitation interventions in persons with multiple sclerosis: A systematic review. Mult. Scler. Relat. Disord..

[14] McFarland S., Coufopolous A., Lycett D. (2021). The effect of telehealth versus usual care for home-care patients with long-term conditions: A systematic review, meta-analysis and qualitative synthesis. J. Telemed. Telecare.

[15] Gagnon M.P., Desmartis M., Labrecque M., Car J., Pagliari C., Pluye P., Frémont P., Gagnon J., Tremblay N., Légaré F. (2012). Systematic review of factors influencing the adoption of information and communication technologies by healthcare professionals. J. Med. Syst..

[16] Shahrabani S., Mizrachi Y. (2016). Factors affecting compliance with use of online healthcare services among adults in Israel. Isr. J. Health Policy Res..

[17] Iacono T., Stagg K., Pearce N., Chambers A.H. (2016). A scoping review of Australian allied health research in ehealth. BMC Health Serv. Res..

[18] Gladwell M. (2006). The Tipping Point: How Little Things Can Make a Big Difference.

[19] Houtrow A., Harris D., Molinero A., Levin-Decanini T., Robichaud C. (2020). Children with disabilities in the United States and the COVID-19 pandemic. J. Pediatric Rehabil. Med..

[20] Rabatin A.E., Lynch M.E., Severson M.C., Brandenburg J.E., Driscoll S.W. (2020). Pediatric tele-rehabilitation medicine: Making your virtual visits efficient, effective and fun. J. Pediatric Rehabil. Med..

[21] Tanner L.R. (2020). Oncology Tele-Rehabilitation: A Race to Access and Outcomes.

[22] Tanner K., Bican R., Boster J., Christensen C., Coffman C., Fallieras K., Long R., Mansfield C., O’Rourke S., Pauline L. (2020). Feasibility and Acceptability of Clinical Pediatric Tele-rehabilitation Services. Int. J. Tele-Rehabil..

[23] Krasovsky T., Silberg T., Barak S., Eisenstein E., Erez N., Feldman I., Landa J. (2021). Transition to Multidisciplinary Pediatric Tele-rehabilitation during the COVID-19 Pandemic: Strategy Development and Implementation. Int. J. Environ. Res. Public Health.

[24] Steinhart S., Raz-Silbiger S., Beeri M., Gilboa Y. (2020). Occupation Based Tele-rehabilitation Intervention for Adolescents with Myelomeningocele: A Pilot Study. Phys. Occup. Ther. Pediatrics.

[25] Knierim K., Palmer C., Kramer E.S., Rodriguez R.S., VanWyk J., Shmerling A., Holtrop J.S. (2021). Lessons learned during COVID-19 that can move telehealth in primary care forward. J. Am. Board Fam. Med..

[26] Health Insurance Portability and Accountability Act of 1996. Public Law No. 104–191, 110 Stat. 1936 (1996). https://www.govinfo.gov/content/pkg/PLAW-104publ191/pdf/PLAW-104publ191.pdf.

[27] Leichsenring F. (2004). Randomized controlled versus naturalistic studies: A new research agenda. Bull. Menn. Clin..

[28] Clarke V., Braun V. (2017). Thematic analysis. J. Posit. Psychol..

[29] Gürel E., Tat M. (2017). SWOT analysis: A theoretical review. J. Int. Soc. Res..

[30] Crowe M., Inder M., Porter R. (2015). Conducting qualitative research in mental health: Thematic and content analyses. Aust. N. Z. J. Psychiatry.

[31] Cottrell M.A., Galea O.A., O’Leary S.P., Hill A.J., Russell T.G. (2017). Real-time tele-rehabilitation for the treatment of musculoskeletal conditions is effective and comparable to standard practice: A systematic review and meta-analysis. Clin. Rehabil..

[32] Kairy D., Lehoux P., Vincent C., Visintin M. (2009). A systematic review of clinical outcomes, clinical process, healthcare utilization and costs associated with tele-rehabilitation. Disabil. Rehabil..

[33] World Health Organization (2017). Global Diffusion of eHealth: Making Universal Health Coverage Achievable: Report of the Third Global Survey on eHealth.

[34] (2020). Health. Virtually. Everywhere. Policy Principle Paper. American Telemedicine Association. http://ncoil.org/wp-content/uploads/2020/09/Policy-Principles-2020-FINAL.pdf.

[35] Olson C.A., McSwain S.D., Curfman A.L., Chuo J. (2018). The current pediatric telehealth landscape. Pediatrics.

[36] Wong M.Y., Gunasekeran D.V., Nusinovici S., Sabanayagam C., Yeo K.K., Cheng C.Y., Tham Y.C. (2021). Telehealth demand trends during the COVID-19 pandemic in the top 50 most affected countries: Infodemiological evaluation. JMIR Public Health Surveill..

[37] Wiederhold B.K. (2020). Connecting through technology during the coronavirus disease 2019 pandemic: Avoiding “Zoom Fatigue”. Cyber Psychol. Behav. Soc. Netw..

[38] Turolla A., Rossettini G., Viceconti A., Palese A., Geri T. (2020). Musculoskeletal physical therapy during the COVID-19 pandemic: Is tele-rehabilitation the answer?. Phys. Ther..

[39] Hoffman L.C. (2018). Telehealth, children, and pediatrics: Should the doctor make house calls again, digitally. Nova L. Rev..

[40] Sharabi A., Somekh I., Waisman Y. (2020). Advances in telemedicine remote vs conventional physical examination. Emerg. Med. Investig..

[41] Mayer N., Portnoy S., Palti R., Levanon Y. (2021). The Efficacy of Tele-Rehabilitation Program for Improving Upper Limb Function among Adults Following Elbow Fractures: A Pilot Study. Appl. Sci..

[42] Dantas L.O., Barreto R.P., Ferreira C.H. (2020). Digital physical therapy in the COVID-19 pandemic. Braz. J. Phys. Ther..

[43] Seron P., Oliveros M.J., Gutierrez-Arias R., Fuentes-Aspe R., Torres-Castro R., Merino-Osorio C., Nahuelhual P., Inostroza J., Jalil Y., Solano R. (2021). Effectiveness of tele-rehabilitation in physical therapy: A rapid overview. Phys. Ther..

[44] Reich A. (2020). Microsoft donates Xbox adaptive controllers to children at ALYN hospital. Jerus. Post..

[45] Scott Kruse C., Karem P., Shifflett K., Vegi L., Ravi K., Brooks M. (2018). Evaluating barriers to adopting telemedicine worldwide: A systematic review. J. Telemed. Telecare.

[46] Israel Society of Occupational Therapy (ISOT) (2020). Telehealth services in occupational therapy (position paper). IJOT.

